# *Baccharis dracunculifolia* and *Dalbergia ecastophyllum*, Main Plant Sources for Bioactive Properties in Green and Red Brazilian Propolis

**DOI:** 10.3390/plants9111619

**Published:** 2020-11-21

**Authors:** Adela Ramona Moise, Otilia Bobiş

**Affiliations:** 1 Department of Apiculture and Sericulture, Faculty of Animal Breeding and Biotechnologies, University of Agricultural Sciences and Veterinary Medicine Cluj-Napoca, 400372 Cluj-Napoca, Romania; adela.moise@usamvcluj.ro; 2 Life Science Institute “King Michael I of Romania”, University of Agricultural Sciences and Veterinary Medicine Cluj-Napoca, 400372 Cluj-Napoca, Romania

**Keywords:** *Baccharis dracunculifolia*, *Dalbergia ecastophyllum*, propolis, chemical composition, bioactive properties

## Abstract

Nowadays, propolis is used as a highly valuable product in alternative medicine for improving health or treating a large spectrum of pathologies, an ingredient in pharmaceutical products, and also as a food additive. Different vegetal materials are collected by honeybees and mixed with wax and other own substances in order to obtain the final product, called propolis. It is known as the bee product with the widest chemical composition due to the raw material collected by the bees. Different types are known worldwide: green Brazilian propolis (having *Baccharis dracunculifolia* as the major plant source), red Brazilian propolis (from *Dalbergia ecastophyllum*), European propolis (*Populus nigra* L.), Russian propolis (*Betula verrucosa Ehrh*), Cuban and Venezuelan red propolis (*Clusia* spp.), etc. An impressive number of scientific papers already demonstrate the pharmacological potential of different types of propolis, the most important activities being the antimicrobial, anti-inflammatory, antitumor, immunomodulatory, and antioxidant activities. However, the bioactive compounds responsible for each activity have not been fully elucidated. This review aims to collect important data about the chemical composition and bioactive properties of the vegetal sources and to compare with the chemical composition of respective propolis types, in order to determine the connection between the floral source and the propolis properties.

## 1. Introduction

Plants and propolis have a well-defined connection because the honeybees collect different resins from plants, shrubs, and trees, and use it as raw material for the production of this important bee product. Propolis is an important product of the hive, with many uses for humans. It has been used in human and veterinary medicine since ancient times and is characterized by variable chemical composition. This variability is due to the plant sources from where the bees harvest the resins, which makes it different from other natural products derived from medicinal plants [1]. Propolis, also called bee glue, has a dark color, it is a sticky product collected by the bees from living plants, which is mixed with wax and other bodily substances, and used for the construction, adaptation, and hygiene of their nests.

One of the characteristics of this lipophilic material is that it is hard and brittle when cold, and soft, pliable, and very sticky when warm; for this reason, sometimes it is named “bee glue” [2]. It has an aromatic smell, and different colors, depending on its source, period of harvesting, and time of storage in the hive. The main types of chemical substances found in propolis are waxes, resins, balsams, aromatics and ethereal oils, pollen, and other organic compounds [3] and they can vary dependent on the place or district, collection time [4], and most importantly, the botanical source. The compounds identified in propolis [5,6] come from three different sources: plant exudates and resins collected by bees, substances secreted from the bee’s metabolism, and various materials introduced during the propolis production [7].

Propolis possesses many beneficial biological activities: antioxidant, antibacterial, antifungal, antiviral, anti-parasitic, anti-inflammatory, anti-proliferative, antiulcer, local anesthetic, hepatoprotective, antitumor, and immune-stimulatory [1,5,8,9,10].

Different types of propolis are available worldwide: green Brazilian propolis (derived from *Baccharis dracunculifolia*), red Brazilian propolis (*Dalbergia ecastophyllum*), European propolis (*Populus nigra* L.), Birch or Russian propolis (*Betula verrucosa Ehrh*) [11], Cuban and Venezuelan red propolis (*Clusia* spp.), “Pacific” propolis (plant origin unknown), “Canarian” propolis (plant origin unknown), etc. The chemical composition and bioactive compounds are described in many scientific studies [4,7,12]. Most of the literature identifies the geographical origin of propolis [13,14,15], but fewer studies make a connection between the plants providing the resins and the propolis chemical composition and properties. Moreover, the majority of scientific studies only describe the link between the plants and poplar types of propolis (derived from *Populus* spp.), probably because they are the most widespread [6,16,17].

**Figure 1:**
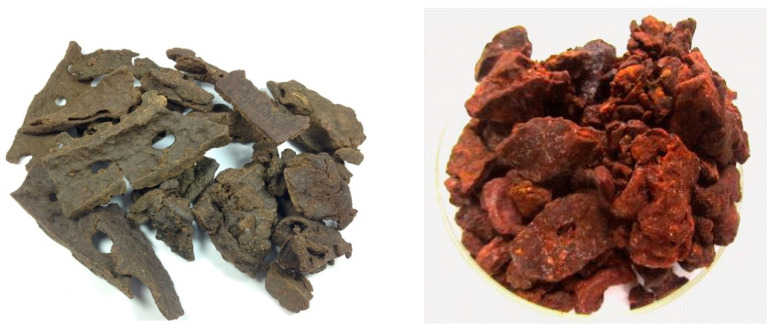
Green and red Brazilian propolis (photo source: [18,19]).

The purpose of this review is to create an overview of the green and red Brazilian propolis characteristics (Figure 1) and to establish the correlation between the plant source (*Baccharis dracunculifolia,* and *Dalbergia ecastophyllum*) and the final product, in order to demonstrate the importance of plant components in expressing the bioactive properties of propolis.

## 2. General Information about Plant Sources of Propolis

Geographical and climatic factors, plant resources, and collection seasons are determined by the specificity of local flora. This determines the classification of the propolis in a certain group [1]. In temperate areas like Europe, North America, or some non-tropical Asian zones, the primary source of propolis is considered the resins of *Poplar* spp. buds. *Poplar* genus contains more species like *Populus alba*, *P. tremula*, and *P. nigra* [20], and the propolis derived from their resins is called poplar or European propolis. Other important sources of European propolis are *Betula pendula*, *Acacia* sp, *Aesculus hippocastanum*, *Alnus glutinosa*, and *Salix alba* [1,21], *Quercus* spp., *Fraxinus* spp., and bark from coniferous trees, such as spruce *Picea* spp., *Abies* spp. or *Pinus* spp. [22,23].

In tropical and subtropical regions, propolis has a different aspect, chemical composition, and properties, due to the difference in vegetation, which make it different from the poplar propolis. *Baccharis dracunculifolia*, *Dalbergia ecastophyllum*, *Araucaria angustifolia*, and *Eucalyptus citriodora* are the main sources of Brazilian propolis; propolis from Venezuela and Cuba are characterized by *Clusia* species (*C. minor*, *C. rosea*), while red Mexican propolis is collected from the *Albergia* genus [21,24,25]. Bees use the secretions of *Xanthorrhoea*, *Acacia*, and *Plumeria* species for propolis production in tropical countries like Australia, North Africa, and Hawaii, respectively [26].

### 2.1. Baccharis dracunculifolia—Source of Green Brazilian Propolis

*Baccharis dracunculifolia* belongs to the Baccharis genus, a Baccharidinae (Asteraceae) subtribe and is widespread in South America, having around 500 species. *Baccharis dracunculifolia* is popularly known as “vassourinha”, “alecrim do campo”, or “alecrim de vassoura”, and is spread across the southern regions of Brazil, but is also in countries like Argentina, Uruguay, Paraguay, and Bolivia [27]. It is a native and widely distributed shrub, that develops rapidly and can be potentially used in soil restoration as a successful colonizer of poor, acid soils. The blooming occurs after the rainy season in the aforementioned countries. Regarding the phenological phases, Baston et al. (2011) [27] shows that the flowering period is from December to May and the vegetative period from June to November; Apis mellifera bees collect resin from the vegetative apices of *B. dracunculifolia*. According to Lima (2006) [28], resin collection visits by honeybees occur from August to April, and in December, January, February, and April harvesting takes place with the highest frequency. Moreover, it is demonstrated [27] that bees collect independently of individual gender (male or female) and its phenological state (flowering or vegetative), and the period of high resin collection visits coincide with the harvest of green propolis. Pollination of the respective trees is mainly carried out by *Apis mellifera* bees [29].

Weinstein Teixeira et al. (2005) [30] compared the chemistries of *B. dracunculifolia* (alecrim) apices, resins, and green propolis and observed differences in the chemical composition of vegetal sources of green propolis. Some of the identified compounds lacked traceability in propolis, and they concluded that other factors besides flora, for example, harvesting season or alecrim phenophase influence propolis chemical composition. Other authors [31,32] obtained similar results and suggested that propolis composition can be influenced also by other factors. Weinstein Teixeira et al. (2005) [30] claim that the integer apices collected for chemical analyses were chemically different from the apices visited by the bees and were converted into resin masses.

An experiment aiming to understand the plant-insect interaction [33] was developed recently; 24 females and 24 male plants were analyzed, as well as the chemical composition of their leaves and the propolis coming from those. According to the results presented by the authors, the male plants showed higher infestation by galling insects, while females registered a higher number of visiting bees, time of resin collection, and terpenes concentration. The obtained results suggested that increasing the percentage of female *B. dracunculifolia* plants may attract more bees and in this way, increasing the production of green propolis.

The leaf extracts of *Baccharis dracunculifolia* has an anti-inflammatory, antibacterial, immunomodulatory, antigenotoxic, and antimutagenic effect [34,35,36,37]. The major compounds separated from this plant are baccharin, artepillin C, and coumaric acid; the studies demonstrated that they are responsible for anticancer effects, especially against breast and prostate cancer [38,39,40,41,42,43].

Stress, smoking, nutritional deficiencies, and ingestion of non-steroidal-anti-inflammatory drugs are some factors that facilitate the appearance of ulcers. *B. dracunculifolia* is the main source of green propolis widely used in Brazilian folk medicine for the treatment of inflammation, hepatic disorders, and stomach ulcers [36].

Many phytochemical studies [34,35,36,44] demonstrated the great variability of compounds in *B. dracunculifolia*; those include flavonoids (isosakuranetin, aromadendrin-4′-methyl ether), terpenes (baccharin), and phenolic acids (artepelin C, caffeic acid, p-coumaric acid, ferulic acid) and are known to be responsible for the majority of the propolis effects.

Lemos et al. (2007) [37] evaluated the possibility of using hydroalcoholic extract obtained from aerial parts of *B. dracunculifolia* for antiulcer treatment. The results showed that doses of 50, 250, and 500 mg/kg of *B. dracunculifolia* crude extract significantly diminished the lesion index, the total lesion area, and the percentage of the lesion compared with negative control groups. Using the model of gastric secretion, the study revealed reductions in the volume of gastric juice and total acidity, as well as the increase of the gastric pH. These results led the authors to conclude that *B. dracunculifolia* could be a potential ingredient in phytotherapeutic preparations for the treatment of gastric ulcers.

### 2.2. Dalbergia ecastophyllum—Source of Red Brazilian Propolis

In Brazil, many types of propolis are distinguished by their botanical origin. Here, the bees collect propolis all year. Bueno-Silva et al. (2016) [45] analyzed that red Brazilian propolis had as a plant source *Dalbergia ecastophyllum*. The effect of the propolis time collection, its chemical composition, and antibacterial activity was examined by these authors and their results demonstrated that the season of harvesting had an important effect on propolis chemical composition, and as a consequence, properties like the antibacterial activity are also influenced. *Dalbergia ecastophyllum* (L) Taub. (Leguminosae) is intensively visited by the honeybees in order to collect red resinous exudates from the branches. It is popularly known as “rabo-de-bugio”, and traditionally its roots and barks are used for uterine inflammation and anemia treatment [46].

Vieira de Morais et al. (2018) [47] claim that recently, the populations of *D. ecastaphyllum* have shrunk considerably, due to anthropic actions including burning and deforestation. The literature is very poor in information on the chemical composition of the resinous exudates of the plant, and from the literature collected literature, we found the research of the above-mentioned authors contained valuable information about the hydro-ethanol extracts of *D. ecastaphyllum* (L) Taub. from Brazil. The study contained data about the contents of total phenols, flavonoids, carotenoids, and chlorophylls, as well as antioxidant, photoprotective, and inhibitory activity of tyrosinase. The total phenolic and flavonoid content of leaf extracts was determined spectrophotometrically, and the results were expressed in Gallic acid equivalents (GAE) per g of dry weight and in mg Quercetin equivalents (QE) per gram of dry weight, respectively. Moreover, for the separation and quantification of phenolic compounds, an HPLC system was used, equipped with SPD-M20A photodiode array detector (PAD). Generally, the total phenolic content ranged between 297.89 and 378.43 mg GAE/g, and the total flavonoid content from 10 to 28.59 mg EQ/g. Data obtained by HPLC showed the separation of eleven phenolic compounds: five belong to the phenolic acids group: caffeic (0.26–0.29 mg/L), sinapic (0.50–1.28 mg/L), vanillic (0.90–2.19 mg/L), protocatechuic (1.13–2.53 mg/L), and β-resorcylic acids (6.72–22.12 mg/L), another six phenolic compounds identified belong to the flavonoids group including four different classes: flavanols (catechin and epicatechin, in a range between 1.56 to 28.03 mg/L and 0.65–1.35 mg/L, respectively), flavonols (quercentin 0.93–0.98 mg/L), flavanones (naringin and naringenin 1.28–3.73 mg/L and 0.26–1.39 mg/L), and flavones (rutin 4.83–20.23 mg/L).

Two different methods were used for antioxidant activity determination: free radical scavenging capacity (DPPH), which is a test based on the reaction transfer of hydrogen atoms, and inhibition of β-carotene bleaching, which is based on the electron transfer reaction. The percentage evaluated by the inhibition test of β-carotene bleaching was lower than the inhibition determined by the DPPH method. This last method indicated results identical to that obtained with quercetin, suggesting the high antioxidant potential of *D. ecastaphyllum* extracts, although not comparable with the antioxidant activity of gallic acid.

A recent study [48] analyzed the chemical composition of *D. ecastaphyllum*, the main source of red Brazilian propolis. The results confirmed that its resins contain liquiritigenin, isoliquiritigenin, formononetin, vestitol, neovestitol, medicarpin, and 7-O-neovestitol. Those findings confirmed the results obtained earlier [49], where red propolis and *D. ecastaphyllum* extracts were analyzed by HPLC.

Another recent study [50] evaluated and compared the chemical composition, antioxidant activity, and microbiological quality of extracts obtained from stem and leaf samples of *D. ecastaphyllum*. Gas-liquid chromatography with flame ionization detection (GC-FID) was used for fatty acid profile determination, total phenolics and flavonoids were determined spectrophotometrically, following consecrated methods. Chemical compounds from the class of polyphenols were determined using liquid chromatography-mass spectrometry detection (LC-MS). The lipid composition comprised of different types of methyl esters, saturated fatty acids, polyunsaturated fatty acids, and monounsaturated fatty acids. The chromatographic profile determined by LC-MS allowed the identification of 49 compounds (phenolic acids and flavonoids).

The difference in some compounds of red propolis and *D. ecastaphyllum* extracts, suggests the contribution of different botanical plant sources for Brazilian red propolis.

Even if the literature is poor regarding the *D. ecastaphyllum* chemical composition and its beneficial effects, some of the studies demonstrate the ability to inhibit the tyrosinase activity and it has a good protective effect, and the above-mentioned study demonstrates its potential for use in the cosmetic and pharmaceutical industries.

## 3. Propolis Chemical Composition

### 3.1. Main Classes of Compounds

The chemical composition of propolis is directly influenced by the resins and balms of the plants from which it comes. The development of new and modern methods permitted the identification and in some cases the quantification of over 300 chemical compounds in propolis [11].

Raw propolis contain generally 50% plant resins, 30% waxes, 10% essential and aromatic oils, 5% pollen, and 5% other organic substances [4]. The main chemical groups present in propolis resin comprise phenolic acids and their esters, flavonoids (flavones, flavanones, flavonols, dihydroflavonols, and chalcones), terpens, aromatic aldehydes, alcohols, fatty acids, stilbenes, and β-steroids [51].

Phenolic acids and their esters represent an important class of chemical compounds in propolis; one of the most important and studied phenolic acids in propolis is caffeic acid phenethyl ester (CAPE) [51], but phenolic acids are a very large group, as described by different studies [26,30,37].

The most important flavonoids in propolis are flavones (luteolin), flavonols (quercetins and derivatives), flavanones (pinocembrin or 5,7-dihydroxyflavone and its derivatives and naringenin), flavanonols (garbanzol and alnustinol), chalcones and dihydrochalcones, isoflavones (calyco-sin), isodihydroflavones (daidzein), flavans, isoflavans (vestitol and its derivatives), and neoflavonoids (homopterocarpin and medicarpin) [26]. Those are responsible for the main pharmacological effects, they can range between 6.2 and 18.8% [11] and their activities will be discussed in the following sections.

**Figure 2:**
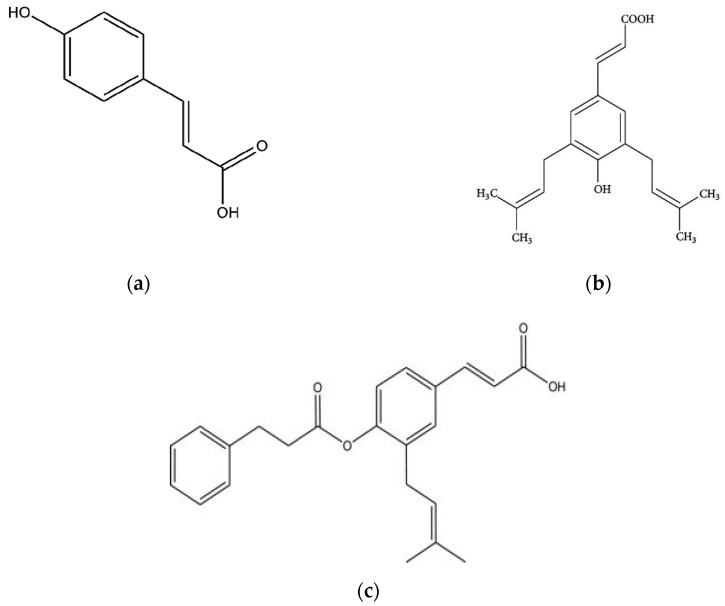
Chemical structure of the main green Brazilian propolis markers (**a**) para-coumaric acid; (**b**) artepillin C; (**c**) baccharin.

Para-coumaric acid, artepillin C, and baccharin are three compounds, separated from green Brazilian propolis as well as from its plant source [33] and are considered as chemical markers (Figure 2); they were quantified in *B. dracunculifolia* leaves, as well as in green propolis hydroalcoholic extracts. The authors reported for the first time 15,16-epoxy-19-hydroxy-1,3,13(16),14-clerodatetraen-18-oic acid (terpens class), and *E*-baccharin 5″-aldehyde (phenolic compound) in both green Brazilian propolis and *B. dracunculifolia* leaves. Moreover, a comprehensive review on artepillin C, a diprenyl-p-hydroxycinnamic acid derivative, responsible for gastroprotective, anti-inflammatory, antimicrobial, antioxidant, and antitumor propolis effect was made recently [52].

**Figure 3:**
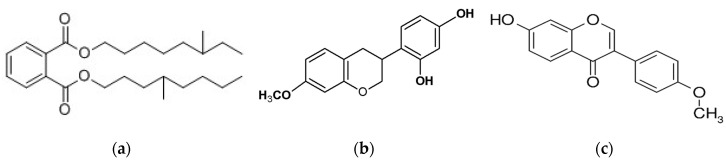
Chemical structure of the main red Brazilian propolis markers (**a**) vestinol; (**b**) neovestinol; (**c**) formononetine.

Aromatic acids are represented by ferulic, cinnamic, caffeic, benzoic, salycilic, and p-cumaric acid. Bueno-Silva et al. (2016) [45] stated that in Brazilian red propolis and resin, formononetin is the most abundant compound, while isoliquiritigenin, (3S)-neovestitol, and (3S)-vestitol are suggested to be responsible for its antimicrobial activity (Figure 3).

The terpenes are volatile components of the plant and red Brazilian propolis are represented by terpineol, camphor (monoterpenes), ferruginol, junicedric acid, pimaricacid, totarolone (diterpenes), upeol, lanosterol, amyrone and derivatives (triterpenes), γ-elemene, valencene, α-ylangene, and α-bisabolol (sesquiterpenes), and together with the phenolic compounds, they imprint the characteristic smell of propolis [13,53].

Trucheva et al. (2006) [54] reported the identification of new constituents in red Brazilian propolis and demonstrated that independently of its plant source and chemical composition, it always possesses antimicrobial and antioxidant activity. The authors separated 14 different compounds, mainly simple phenolics, triterepenoids (α-amyrin, β-amyrin, cycloartenol and lupeol, ketone 20(29)-lupen-3-one), isoflavonoids (isoflavan isosativan and pterocarpan medicarpin), prenylated benzophenones, and a naphthoquinone epoxide.

Compared to green or brown propolis, the red Brazilian propolis is rich in polyphenols, more than 30 different phenolic compounds have been identified so far [9,46,53] and more than 300 components from different chemical classes have been identified [55]. Unlike other types of propolis, red propolis has a specific chemical composition presenting some compounds never before reported in other propolis types, such as vestitol and neovestitol, biochamine A, as well as liquiritigenin, formononetine, and medicarpine [2].

### 3.2. Identification and Quantification Methods of the Main Propolis Compounds

An important role in the identification and quantification of propolis compounds is attributed to the extraction methods. The highest content of antioxidant compounds is obtained when the extraction is made with ethanol. For some compounds such as artepillin C or p-coumaric acid, the supercritical fluid extraction with CO_2_ is more efficient [9].

Weinstein Teixeira et al. (2005) [30] used Gas Chromatography (GC)/Electron Ionization Mass Spectrometry (EIMS) for the identification of different chemical compounds of green Brazilian propolis samples, male and female *B. dracunculifolia* apices, and resin masses. Vegetal and resin materials were extracted with methanol. In the same paper, the authors found that the bee’s preference for the apices is mostly for the female plants (55.9%) and only 44.1% for the male plants, but often, integer and robust apices were refused, and the bees moved on to other *B. dracunculifolia* plants; as this behavior is not fully understood, the authors believe that some of the volatile substances (from resiniferous ducts or glandular trichomes) are probably more effective in attracting bees for resin collection.

Phenolic compounds in shoot apices of *B. dracunculifolia* (*Asteraceae*), resin masses from bee’s corbiculae, and green propolis demonstrated the presence of (i) cinnamic acid derivatives, (ii) prenylated cinnamic acid derivatives, (iii) chromane derivatives, (iv) naphtalene and anthracene derivatives, and (v) simple benzene and phenol derivatives [30]. While some cinnamic acid derivatives such as (i) hydrocinnamic acid, p-hydroxycinnamic acid, p-methoxycinnamic acid, and trans-3-methoxy-4-hydroxy-cinnamic acid were presented in all apices, resin, and propolis samples, some others like p-hydroxyhydrocinnamic acid were presented only in propolis, but not in *B. dracunculifolia* apices and resins. Others, like p-coumaric acid, certainly come from resins as it was found here only in propolis, not in apices. The majority of prenylated cinnamic acid derivatives that were in green Brazilian propolis from resins included (ii) allyl-3-prenylcinnamate, 4-hydroxy-3-prenylcinnamic acid, and 4-hydroxy-3,5-diprenylcinnamic acid (also known as artepillin C), but only allyl-3-prenylcinnamate was present in male and female apices. The most representative compounds of chromane derivatives were (iii) 2,2-dimethylchromene-6-propenoic acid and 2,2-dimethyl-8-prenylchromene-6- propenoic acid; those were detected in all analyzed samples of apices, resins, and propolis. A different outcome was registered for 8-(methyl-butanechromane)-6-propenoic acid, which was not present in apices or resins, but only in propolis. The resins of *B. dracunculifolia* were the source of 2-t-Butylnaphto-[2,3-b]-furan-4,9-dione, 2-hydroxy-7,12-dimethyl-benzanthracene, and 1-hydroxyl-2-(1-methoxyethyl)-3-methoxyanthraquinone (class iv). Some of the phenol derivates (v) had different origins in green Brazilian propolis: p-vinylphenol was not presented in apices or resins, but it was determined in propolis, while p-vinyl-o-prenylphenol was presented in all tree samples types [30].

Phenolic compound as 3-prenyl-4-hydroxycinnamic acid (PHCA), 2,2-dimethyl-6-carboxyethenyl-2H-1-benzopyrane (DCBEN), 3,5-diprenyl-4-hydroxycinnamic acid (DHCA), and 2,2-dimethyl-6-carboxyethenyl-8-prenyl-2H-1-benzopyran (DPB) were identified in Brazilian propolis by mass spectrometry (MS) and nuclear magnetic resonance (NMR) techniques [56]. For identification and quantification of the 3,5-diprenil-4-hidroxicinamic (Artepillin C) and acid 4-hidroxicinamic (p-coumaric acid) in the propolis extracts, High Performance Liquid Chromatograph EZChrom Elite equipped with diode detector was used [9].

Using the same GC method, other authors [30] investigated the presence of terpenoids in the same three types of samples. The results showed that some class representatives presented in apices, were not founded in green Brazilian propolis; at the same time, some of them (stigmasta-3,5-dien-7-one, cholest-5-en-3β-ol, and clionasterol) were presents in resins, but not in green propolis. Gas chromatography analysis developed by de Andrade de Carvalho et al. (2020) [57], revealed the presence of hydrocarbons, alcohols, ketones, ethers, and terpenes, such as lupeol, lupenone, and lupeol acetate, in red propolis extracts.

Trusheva et al. (2006) [54] using repeated column chromatography on silica gel with n-hexane—acetone gradient, isolated 20 different fractions, which were further subjected to GS/MS. A very nonpolar fraction in red Brazilian propolis was identified by GC-MS and was composed of five phenylpropene derivatives: trans-anethol, methyl eugenol, trans-methyl isoeugenol, elemicin, and trans-isoelemicin. Those components seemed to be responsible for the particular anis-smell of the red Brazilian propolis.

Mendonça-Melo et al. (2017) [58] presented chemical and genetic similarities between *D. ecastaphyllum* and red propolis from Northeastern Brazil. Ethanolic extracts of *D. ecastaphyllum* bark samples were prepared and the HPLC system with DAD detector and the UHPLC Acquity chromatographer coupled with a TQD Acquity mass spectrometer were all applied in order to characterize the samples. The HPLC chromatograms obtained by these authors showed that propolis compared with the plant species presented similarity in some peaks, but the number and intensity of obtained peaks were much elevated in propolis samples. ESI -MS fingerprint of *D. ecastaphyllum* samples revealed similar composition profiles, and propolis samples presented ions which are also found in *D. ecastaphyllum*, but some others were highlighted only in propolis. This study allowed the identification of pinocembrine, biochanin A, daidzein, and formononetin in propolis, their origin already attributed to the vegetal source of this propolis.

## 4. Bioactive Properties of Green and Red Propolis Due to the Chemical Composition

### 4.1. Antioxidant Activity

The antioxidant activity of propolis is demonstrated by the majority of outcomes which proved the reduction in oxidative stress markers. The polyphenols, one of the major classes of compounds in propolis, have a chemical structure capable of effectively eliminating free radicals. On the other hand, the flavonoids in propolis are powerful antioxidants, capable of scavenging free radicals and in this way, protect cell membranes against lipid peroxidation [59,60,61].

Using the DPPH (1,1-diphenyl-2-picrylhydrazyl) method [9,54], the antioxidant activity of different compounds isolated from red Brazilian propolis was tested as shown in Table 1; prenylated benzophenones were considered to play an important role in the antioxidant activity of propolis against DPPH.

ABTS (2,2′-azinobis-3-ethylbenzothiazoline-6-sulphonic acid) was another successful method used for the determination of the antioxidant activity. Based on the spectrophotometric measurement, the results were generally expressed as TEAC (Trolox (vitamin E precursor: 6-hydroxil-2,5,7,8-tetramethylchromo-2-carboxilic acid) equivalent antioxidant capacity).

A direct method for antioxidant activity determination was represented by the electron paramagnetic resonance (EPR), which allowed us to record a direct signal from the DPPH free radicals. In contact with propolis, the EPR DPPH signal was extinct and this directly related to the propolis antioxidant properties [66,67]. Moreira Pazin et al. 2017 [68] using the EPR method analyzed the antioxidant activities of propolis produced by different bee species and compared it to the antioxidant activity of green propolis produced by the honey bee *Apis mellifera*, and concluded that the Brazilian green propolis had lower antioxidant activity than propolis types produced by stingless bees.

### 4.2. Antibacterial and Antifungal Activity

The antibacterial activity of propolis is firstly its direct action on microorganisms, and secondly its stimulation effect on the immune system, resulting in the enhancement of the natural defenses in the organisms. The mechanism by which propolis acts on bacteria involves the decreased cell membrane permeability of microorganisms, the decreased membrane potential, ATP production, and the decreased bacterial mobility [11]. The antibacterial activity of propolis is higher for gram-positive bacteria than gram-negative ones. This is because the outer membranes of gram-negative bacteria produce some hydrolytic enzymes, which have the property to break the active compounds of propolis [69].

Propolis shows an important antibacterial and antifungal activity, regardless of its geographic origin; this property is essential for the preservation and maintenance of the hive and is mainly due to complex synergistic effects between the flavonoids, phenolic acids, and their derivatives, which are mainly present in propolis [70,71,72].

Bettencourt et al. (2015) [62] correlated the radical scavenging activity (IC_50_) and Minimal Inhibitory Concentration (MIC) values and the relative content of all identified metabolites, including the total phenolic compounds, presented in green Brazilian propolis, and they concluded that lower IC_50_ and MIC values were associated with higher antioxidant and antibacterial activities (Table 2).

Antibacterial activity of red Brazilian propolis was investigated by Trusheva et al. (2006) [54] against different bacterial strains (*Staphylococcus aureus*, *Escherichia coli*, and *Candida albicans*). The results demonstrated that components like isoflavonoids are efficient in inhibiting the bacteria, especially against C. albicans. The same author identified that prenylated benzophenone had an important activity against *S. aureus*.

According to Dantas Silva et al. (2017) [10] (Table 2), red propolis showed the highest antimicrobial activity among the samples obtained by ethanolic and supercritical extraction methods. The ethanolic red extract exhibited the highest antimicrobial activity against *Enterococcus* sp., *Staphylococcus aureus*, and *Klebsiella* sp. with MIC values of 31.3, 62.5, and 31.3 μgxmL^−1^.

Bueono-Silva et al. (2013) [78], analyzing the red Brazilian propolis states that the highest antibacterial activity is registered during the rainy season (from January to May in Brazil), and in this period the highest concentration of vestitol, neovestitol, and isoliquiritigenin is recorded, too.

Machado et al. (2016) [9] tested the ethanolic extracts of green and red propolis against two strains of *Staphylococcus aureus* (ATCC 33951 and 25923) and *Escherichia coli*. Considering the best antioxidant activities and the highest content of total phenolic acids and flavonoids, the antimicrobial activity was expected to be at high levels. However, a negative correlation between the concentration of phenolics in the extracts and Minimal Inhibitory Concentration MIC was identified. The extracts obtained from the samples of red propolis showed the best antimicrobial activities (Table 2).

Other authors [79] also evaluated the antibacterial and antifungal efficiency of propolis extracts obtained by different extraction techniques and demonstrated that the EtOH extract has the best antimicrobial potential.

Moreover, Koo et al. (2000) [80] tested red and green propolis from different geographical areas of Brazil. They identified differences in the MIC and MBC for each extract in relation to *Streptococcus mutans*, *S. sobrinus,* and *S. cricetus*, and the best results were found for the red propolis coming from the northeast of Brazil. Similar results were obtained later [9].

Bridi et al. (2015) [81] stated that for the standardization of propolis, considering its very large compositional differences, methods like ORAC (Oxygen Radical Absorbance Capacity) and antimicrobial tests, should be considered in setting international quality standards for propolis, regardless of its geographical origin.

### 4.3. Antiviral Activity

Regardless of its origin, propolis from different geographical regions has considerable antiviral activity by acting at different levels and interfering with the replication of some viruses, like herpes simplex types 1 and 2, adenovirus type 2, influenza virus, or human immunodeficiency virus (HIV) [5,7,12,82,83,84,85,86,87].

The majority of flavonoids (flavonols and flavones) demonstrated their antiviral activity, especially against HSV-1 [83]. The same authors analyzed the effect of propolis on DNA and RNA viruses including HSV-1, HSV-2, adenovirus type 2, vesicular stomatitis virus (VSV), and poliovirus type 2. At a concentration of 30 µg/mL, propolis reduced the titer of herpes virus and vesicular stomatitis virus and at the same time, adenovirus was less susceptible.

Serkedjieva et al. (1992) [88] used isopentyl ferulate, isolated from a Brazilian propolis extract, and demonstrated the suppression of influenza virus A/Hong Kong/1/68 (H3N2) reproduction in vitro.

The adjuvant capacity of green propolis (5 mg/dose) associated to inactivate the Suid herpesvirus type 1 (SuHV-1) vaccine was evaluated [89]. The mice inoculated with SuHV-1 vaccine plus aluminum hydroxide and propolis extract presented higher levels of antibodies. The use of SuHV-1 vaccine plus propolis alone did not induce significant levels of antibodies. However, the combination was able to increase the cellular immune response, evidenced by the increase in the expression of mRNA to IFN-γ. Besides, propolis increased the percentage of protected animals when challenged with a lethal dose of SuHV-1, resulting in the authors concluding its usefulness in vaccines as an adjuvant. Using a mouse model, [90] propolis was added as an adjuvant to inactivated swine herpesvirus type 1 vaccine; the results showed that this bee product stimulated the cellular and humoral increasing IFN-γ.

The influence of green propolis extracts having *B. dracunculifolia*, *B. erioclada*, and *Myrceugenia euosma* as the main vegetal origin were tested [91] on influenza A/PR/8/34 (H1N1) virus propagated Madin-Darby canine kidney (MDCK) cells and female DBA/2 Cr mice. The observations showed the reduction of body weight loss of infected mice and the virus yield in the broncho-alveolar lavage fluids of lungs.

Continuing the research, the same authors [92] tested three types of green Brazilian propolis harvested in different areas of Brazil. These extracts were examined for their anti-HSV-1 efficacies in a cutaneous HSV-1 infection model in mice. The authors concluded that the three ethanol extracts of propolis moderately alleviated the symptoms of cutaneous herpetic infection.

Because it induces an earlier immune response and provides a longer protection period, propolis has been tested as a vaccine adjuvant, mainly due to its flavonoids content, which is a potential adjuvant, enhancing IgG, IL-4, and IFN-γ in serum [93,94].

Fernandes et al. (2015) [95] demonstrated the positive effect of propolis against canine coronavirus. Results show that IFN-γ is an effective way to measure the cellular response induced by a vaccine.

### 4.4. Anti-Parasitic Activity

Regueira-Neto et al. (2018) [96] analyzed the antiparasitic effect of the resin obtained from *Dalbergia ecastophyllum* and compared its activity with those of red Brazilian Propolis. They used the samples against *Leishmania* and *Trypanosoma* parasites. The results obtained showed that generally, propolis samples demonstrated a better performance against the parasites when compared to the *D. ecastaphyllum* resin extract. This result suggests that the honeybees modulate the chemical compounds present in the plant resins when they mix the plant material with their own secretions during the production of red propolis. Other representative results of the same study demonstrated once again the effect of seasonality on red propolis samples: the red propolis collected during the rainy season showed to be more effective than the one collected in the dry season.

In vitro activity of ethanolic red and green Brazilian propolis extracts against *Trypanosoma cruzi* (Y strain) epimastigotes were tested [10]. This protozoan parasite causes American trypanosomiasis and is widespread in Latin America. The authors stated that ethanolic propolis extracts inhibited the growth of *T. cruzi* epimastigote cultures at concentrations of 75 and 300 mg/mL, generally, all propolis samples demonstrated high inhibitory activity against *T. cruzi* compared to the control group. More than that, the results demonstrated that one of the red propolis extracts showed the highest activity, leading to 98% inhibition of growth in 24 h of incubation. These results come to confirm other previous studies [97].

### 4.5. Anti-Inflammatory Activity and Wound Healing Effect

Inflammation occurs in response to the constant exposure to environmental and endogenous stimuli as well as to accidental damage. Wound healing is a dynamic and complex process of skin repair, which occurs in response to an injury. The inflammation represents its first step, followed by reepithelization and remodeling. Once modern methods developed, propolis was tested for its anti-inflammatory effects by many studies [78,87,98,99], and its effect has been demonstrated, moving it from its empirical use to use based on scientific evidence.

Vestinol and neovestinol are two isoflavonoids compounds involved in the anti-inflammatory and immunomodulatory properties of the red propolis [78]. Using neutrophil migration assay, the authors showed that these compounds inhibited neutrophil migration at a dose of 10 mg/kg.

Specific molecular mechanisms behind the anti-inflammatory effect of red propolis were demonstrated [99]. The authors stated that treatments with red propolis extracts, rich in poly-phenolic compounds, reduced the lesion areas in mice, as well as neutrophil infiltration (through the reduction of neutrophils chemotaxis), expression of the major inflammatory transcriptional factor (NF-kB), and the synthesis of inflammatory mediators. Using 12 two months old Swiss male mice, a daily dose of 100mg/kg red propolis extract was applied on the full-thickness excisional wound. Histological analyses were performed for estimating the density of inflammatory cells and count of blood vessels in granulation tissues; moreover, TGF-β, IL-13, TNF-α, IL-6 plasma levels were measured with ELISA assay. Eight days after lesions, the treated group presented better healing; the wound-closure process was improved in the treated mice, with a similar amount of fibroblasts; the treated group presented lower IL-6 and TNF- α levels.

The effects of 13% aqueous extract of propolis were studied [100] as a therapeutic adjuvant for patients with mild to moderate asthma. This study demonstrated that patients receiving propolis daily for two months showed a marked reduction in the incidence and severity of nocturnal attacks and improvement of ventilator functions, which was associated with decreases of prostaglandins, leukotrienes, pro-inflammatory cytokines (TNF-α, IL-6, IL-8), and increased IL-10.

Hori et al. (2013) [98] demonstrated the efficiency of green propolis extract in reducing the IL-1*β* secretion in mouse macrophages and this reduction was correlated with a decrease in the activation of the protease caspase-1. The same authors found that the extract (30 μg/mL) was not toxic to the cells even after 18-h of treatment. These valuable data indicate that Brazilian green propolis extract, rich in Artepillin C, has a role in regulating the inflammasomes (a large molecular platform formed in the cell cytosol in response to stress signals, toxins, and microbial infections).

### 4.6. Antitumor and Anti-Proliferative Activity

Propolis is used as a complementary therapy for cancer treatment. It has shown efficacy against various types, including bladder, blood, brain, breast, colon, head, and neck, kidney, liver, pancreas, prostate, and skin cancers [101].

For the Brazilian green propolis, considerable evidence exists of anticancer properties [102,103]. This type of propolis is not patented, but some of its components were isolated and synthesized, and are now patented drugs for cancer treatment [104].

The literature includes information about the antitumor and anti-proliferative activity of propolis, especially the cytotoxic action of propolis in vitro. It was demonstrated that propolis may have a direct effect on different tumor cells in vitro, and the administration of propolis to animals or humans depends on its solubility and systemic bioavailability. Thus, the antitumor activity of propolis may occur mainly due to its immunomodulatory action, exerting either chemo-preventive or therapeutic effects. However, effective immunotherapy based on propolis has not yet been developed for any type of malignancy. Moreover, tumors have developed numerous mechanisms to evade innate and adaptive immunity. Thus, propolis and its compounds need to be further explored regarding antitumor and immunomodulatory action in vivo [51].

Early in 1995, Matsuno [105] isolated an active substance from Brazilian propolis and characterized it as a new clerodane diterpenoid; this compound inhibited the growth of hepatoma cells and arrested the tumor cells at the S phase.

In vitro activity of the ethanolic extracts of red and green Brazilian propolis was tested on the tumoral cells B16F10 [9], evaluating the anti-proliferative effect. The extracts concentration was 50 and 100 μg/mL, respectively, and the cellular proliferation was measured after 24 and 48 h (Table 3). In both determinations, all extracts showed significant inhibition of cellular proliferation; the best results were shown by the extracts derived from the red propolis from the northeast. The green propolis had a lesser antitumor effect than the red one, but good results were obtained for the sample originating from Parana–Brazil. The same extract of green propolis registered the highest concentration of artepillin C and *p*-coumaric acid. Other important results regarding artepillin C were found by Kimoto et al. (2001) [106], who demonstrated its anti-leukemic effect.

Results obtained by Machado et al. (2016) [9] were in line with those obtained by Franchi-Jr et al. (2012) [110] when they identified that in vitro cytotoxic activity of ethanolic extracts of red propolis against strains of human leukemic cells were superior when compared to the extracts of green propolis.

The inhibitory effect of caffeic acid phenethyl ester (CAPE) on angiogenesis, tumor invasion. And pulmonary metastatic capacity of CT26 cells was demonstrated by Liao et al. (2003) [111]. CAPE also prolonged the survival of mice implanted with CT26 cells, demonstrating its potential as an antimetastatic agent. Concentrations between 10–400 μM CAPE had a dose-dependent effect on the cytotoxicity of C6 glioma cells, reducing the viability to 42% in relation to control, and increasing the proportion of hypodiploid DNA, as an indication of apoptosis [112]. Continuing the research on CAPE, the same authors [113] investigated later its effect on oral cancer using a cultured cancer cell line (squamous cell carcinoma, SAS; oral epidermoid carcinoma-Meng 1, OEC-M1) and normal human oral fibroblast (NHOF). The study contained results regarding the effects on the cell growth pattern, their cytotoxicity, and changes in the cell cycle. CAPE demonstrated cytotoxic effects on tumor cells but not on the NHOF cell line. Flow cytometric analysis showed OEC-M1 cell arrest at the G2/M phase. The authors concluded that the different effects on cancer and normal cells suggested these compounds might be useful in oral cancer chemotherapy.

Generally, the best antiproliferative effect is demonstrated by the red propolis extract, when compared to other samples of propolis; this effect may depend on its differentiated composition. An example in the case of biological activities is the presence of formononetin, which belongs to the isoflavones group, and can only be found in red Brazilian propolis. Moreover, other compounds seem to play an important role in propolis antitumor activities; such as polyisoprenylated benzophenone (xanthochymol), xanthochymol, and formononetin [49,109,114].

As we stated before, the composition of propolis is very complex, and for this reason, more compounds should be investigated in tumor assays in vitro and in vivo, as well as the synergistic effects between them.

### 4.7. Immunomodulatory Action

The immunomodulatory action of propolis depends on its dose, chemical composition, and main components, as well as on the assay conditions. Many in vivo and in vitro studies were developed during the years, especially after the 1900s, demonstrating its stimulant action on the lytic activity of natural killer cells against tumor cells, and on antibody production [69].

Orsolic and Basic (2003) [115] stated that due to its immunomodulatory effect, propolis was used for the treatment of many immune disorders. The effects on macrophages have been demonstrated by [116] since 1999. In the same period, the effect of increasing the ratio of CD4^+^/CD8^+^ T-cells in vivo in mice was studied and demonstrated [117].

Propolis effect on macrophage activation by oxygen (H_2_O_2_) and nitrogen (NO) was evaluated by metabolite determination [118]. The possible co-stimulant activity of propolis (5, 10, and 20 µg/mL) associated with IFN-γ, on H_2_O_2_ and NO production was determined in vitro. As a result, propolis induced an elevation in H_2_O_2_ production (but not statistically significant), suggesting that it may activate macrophages with a consequent oxygen metabolite liberation and interferon-gamma (IFN-γ) being a potent stimulus for macrophage activation. The research also found in mice cell cultures treated with a 250 and 500 µg/mL hydro-alcoholic solution of propolis, activated with IFN-g, a higher H_2_O_2_ release than in non-activated cells. When the animals were treated with 250, 500, and 1000 µg/mL hydro-alcoholic solution of propolis, macrophages stimulated NO production. Inhibition was registered at 3000 and 6000 µg/mL.

Later studies [119] that investigated the growth and metastatic potential of a transplantable mammary carcinoma (MCa) in mice under a water-soluble derivative of propolis (WSDP), caffeic acid (CA), caffeic acid phenethyl ester (CAPE), and quercetin (QU), came to confirm those reported previously [118]. All the analyzed compounds could be potentially useful in the control of tumor growth and moreover, the antitumor activity of tested compounds can be related to their immunomodulatory properties.

Immunomodulation potentials of propolis are well described by Al-Hariri (2019) [120] in a review containing outcomes from 1997 until 2018, about immunomodulatory agents and their potential mechanisms.

### 4.8. Other Biological Activities

Besides the above mentioned biological activities, green and red Brazilian propolis has other important effects in human and animal disease treatments or symptoms amelioration.

The gastric protective effect and anti-ulcer activity of the hydro-alcoholic extract of Brazilian green propolis were demonstrated [121], using models of acute gastric lesions induced by ethanol, indomethacin, and stress in rats. Animals pretreated with propolis hydro-alcoholic crude extract (50, 250, and 500 mg/kg) showed a significant reduction in lesion index, the total affected area, and the percentage of the lesion, the results of which are similar to those obtained for vegetal *B. dracunculifolia* extracts [37]. At the highest tested dose (500 mg/kg), green propolis extract demonstrated significant anti-ulcer protection by reducing the evaluated parameters in the gastric ulceration. Pretreatment of 250 and 500 mg/kg green propolis extract displayed an anti-secretory activity, by reducing the gastric juice volume, total acidity, and pH. All these results suggest that *B. dracunculifolia* leaves extracts, as well as Brazilian green propolis, displays good anti-ulcer activity, their incorporation in ulcer treatment products being fully possible after its pharmacological validation.

The antiulcer activity of red propolis extracts was tested recently [122]. Using anti-inflammatory drug-induced models of rat ulcers, the authors found that the administration of hydroalcoholic extracts of red propolis and formononetin (an isoflavonoid, normally found in red propolis) to pylorus ligature models significantly decreased gastric secretion volumes and increased mucus production. The same study revealed the anti-*Helicobacter pylori* activities of red propolis.

Regarding its local anesthetic effect, propolis is used for the treatment of oral diseases in terms of its antimicrobial activity. The dentistry application of propolis is practiced nowadays in many countries and is subsequently the best scientifically documented area, due to its use in different dental specialties including periodontology, oral mucosa pathology, oral surgery, orthodontics, and prosthodontics [123].

Brazilian propolis has been reported to have hyperglycemic and hyperlipidemia effects [124]. Other studies [125] revealed that Brazilian propolis prevented a high-fat and fructose diet-induced hyperlipidemia, reduced hepatic sterol regulatory element-binding protein (SREBP), but no differences were observed in hepatic PPAR-α and CYP7A1. Chen et al. (2018) [126] analyzed the results obtained by Li et al. (2012) [127] which compared the hyperglycemia effects of Taiwanese green propolis extract and Brazilian propolis extract in T2DM rats. The obtained results obtained showed that treatments with Taiwanese green propolis extract (8 weeks, 183.9 mg/kg/day) and Brazilian propolis extract (10 weeks, 200 mg/kg/day) improved fasting blood glucose levels by 48% and 19%, respectively. These results conducted the authors to conclude that Taiwanese green propolis has better effects on lowering fasting blood glucose than Brazilian propolis extract and appears to regulate hepatic lipid metabolism via a different pathway from Brazilian propolis.

Moreover, scientific demonstrations exist about the inhibitory effect of green Brazilian propolis on acetic acid-induced pain and increasing the pain threshold against infrared and formalin tests developed in a pain model in rats. Promising results regarding the anti-nociceptive and anti-inflammatory properties of green propolis were obtained recently [128], these results indicated that this bee product has to be subjected to future analyses.

The effect of Brazilian propolis was demonstrated for sneezing and nasal rubbing in experimental allergic rhinitis of mice [129]. Significant inhibition on antigen-induced nasal rubbing and sneezing was observed after repeated administration of 1000 mg/kg of Brazilian propolis extract for 2 weeks. The same authors stated that propolis significantly inhibited histamine release from rat mast cells induced by antigen, based on these results, they concluded that propolis may be effective in the relief of symptoms of allergic rhinitis.

## 5. Conclusions

From a chemical point of view, propolis is the main complex bee product; its chemical composition is directly influenced by the vegetal source of the resins, some studies demonstrated a qualitative composition similarity between them. The main studies presented in this review agreed that the propolis samples presented significant differences between them, according to their origin.

This review demonstrated the large diversity of Brazilian propolis, the most known and valuable types being red and green ones; and not only is the great Brazilian biodiversity the cause of the variability between samples but their differing chemical composition results in a different expression of their biological activities. Regardless of their origin, propolis remains an important matrix for further studies in the biomedical domain, because it has demonstrated significant antioxidant, antibacterial, antifungal, anti-viral, anti-parasitic, and anti-tumor effects. The connection between plant resins, exudates, and propolis have been fully demonstrated, all studies showed the higher activity of propolis samples compared to plant extracts, due to the various components added by the bees to produce the final product (propolis).

## Figures and Tables

**Table 1:** Total polyphenolic and flavonoid content and antioxidant activity of red and green Brazilian propolis.

Propolis Type	Samples Data	Analytical Method/Unit	Values	Reference
Green Propolis	Ethanolic extracts	Total polyphenols (Folin-Ciocalteu method)/mgGAE/g	160.98–181.71	[9]
		Total flavonoids/mgQE/g	25.52–46.80	
		RSA (DPPH method)/IC_50_	31.80–101.45	
		ABST (Trolox method)/%	77.90–86.40	
	Ethanol, hexane, and dichloromethane extracts	RSA (DPPH method)/IC50/μg/mL Total polyphenols (Folin-Ciocalteu method)/mgGAE/g	21.50–78.77 38.85–204.30	[62]
	Hydro-alcoholic extract	Total polyphenols (Folin-Ciocalteu method)/mg g^−1^ Total flavonoids (AlCl_3_method)/ mg g^−1^ RSA (DPPH test)/EC50 (μg mL^−1^)	93.7–149.3 6.0–21.0 17.3–83.60	[63]
Red propolis	Ethanolic extracts	Total polyphenols (Folin-Ciocalteu method)/mgGAE/g Total flavonoids (AlCl_3_ method)/ mg QE/g RSA (DPPH method)/IC50 ABST (Trolox method)/%	198.77–300.36 57.60–58.19 44.29–89.32 98.20–98.50	[9]
	Ethanolic extracts	Total polyphenols (Folin-Ciocalteu method)/mgGAE/g RSA (DPPH method)/IC50	151.55 270.13	[64]
	Ethanolic extracts	Total polyphenols (Folin-Ciocalteu method)/mgGAE/g Total flavonoids (AlCl_3_ method)/ mg QE/g RSA (DPPH method)/IC50	232.00 43.00 57.00	[65]
	Ethanolic extracts	RSA (DPPH method)/% Total polyphenols (Folin-Ciocalteu method)/mgGAE/g	0.7–49.00 157.16–300.36	[54]

**Table 2:** Antibacterial and antifungal activity of red and green Brazilian propolis.

Propolis Type	Analyzed Strains	Analytical Method: Results (μg/mL)	References
Green Propolis	*Staphylococcus aureus* *Enterococcus* sp. *Klebsiella* sp. *Escherichia coli* *Candida albicans*	MIC: 250–1000 MIC: 250 MIC: 500–1000 MIC: >1000 MIC: >1000	[10]
	*Staphylococcus aureus (methicillin-resistant/sensitives)*	MIC90: 123.2–369.5	[73]
	*Staphylococcus aureus* (ATCC 33951 and 25923); *Escherichia coli*	MIC: 200–1600 MBC: 800 1600 MIC: 400–1600 MBC: 400–1600	[9]
	*Bacillus subtilis* (ATCC 6633) *Micrococcus luteus* (ATCC 10240) *Staphylococcus aureus* (ATCC 6538)	MIC: 62.5–500 MIC: 62.5–500 MIC: 125–500	[62]
	*Staphylococcus aureus* *Enterococcus faecalis* *Micrococcus luteus*	MIC: 382–650 MBC: 765–1050 MIC: 1352–1822 MBC: 2972–3643 MIC: 400–435 MBC: 935–1040	[63]
	*E. coli* ATCC 25922 *S. aureus* ATCC 29213 *E. faecalis* ATCC 29212 *E. faecalis* 3199 *E. faecium* 3266	MIC: >1600 MIC: 400 MIC: 1600 MIC: 1600 MIC: >1600	[74]
Red propolis	*Streptococcus mutans*	MIC: 293 MIC: 1172	[75]
	*Staphylococcus aureus* *Enterococcus* sp. *Klebsiella* sp. *Escherichia coli* *Candida albicans*	MIC: 62.5–125 MIC: 31.3–62.5 MIC: 31.3–62.5 MIC: >1000 MIC: >1000	[10]
	*Escherichia coli* *S. aureus* *P. aeruginosa*	MIC: 128–512 MIC: 64–1024 MIC: 512	[76]
	*Streptococcus mutans*; *Streptococcus sobrinus*; *Staphylococcus aureus*; *Actinomyces naeslundii*	MIC: 15.6–125 MBC: 31.2–500	[45]
	*Staphylococcus aureus* (ATCC 33951 and 25923); *Escherichia coli*	MIC: 25–600 MBC: 400–1600 MIC: 400–800 MBC: 800–1600	[9]
	*Pseudomonas aeruginosa* *Bacillus subtilis* *Candida albicans* *Salmonella typhimurium* *Klebsiella pneumoniae* *Enterococcus faecalis* *Escherichia coli* *Proteus mirabilis* *Streptococcus pyogenes*	MIC: 256; MMC: 512 MIC: 256; MMC: 512 MIC: 256; MMC: 512 MIC: 512; MMC: 512 MIC: 512; MMC: 1024 MIC: 512; MMC: – MIC: 512; MMC: – MIC: 512; MMC: – MIC 512; MMC: –	[77]
	*Staphylococcus aureus ATCC 25923* *Staphylococcus mutans UA159*	MIC: 25–50	[65]
	*Staphylococcus aureus*; *Escherichia coli*; *Candida albicans*	MIC: 14–19 MIC: 12–14 MIC: 15–29	[54]

MIC—Minimal Inhibitory Concentration; MIC90—Minimal Inhibitory Concentration killing 90% of the bacteria; MBC—Minimal Bactericide Concentration; MMC—Minimal Microbicidal Concentration.

**Table 3:** Antitumor and anti-proliferative of red and green Brazilian propolis.

Propolis Type	Analytical Method/Samples/Unit	Tumoral Cells	Results	References
Green Propolis	Spectrophotometric ELISA colorimetric assay/Ethanolic extracts /Absorbance units	Murine melanoma cellular strain (B16F10)	0.03–0.11	[9]
	CellTiter 96 Aqueous One solution cell proliferation assay kit/ethanolic extract/ 50% growth inhibition/µg/mL	Normal human prostate epithelial (PrEC) Human prostate cancer cells (RC-58T)	5.5–8.75 3.0–5.5	[107]
	Cell proliferation assay/supercritical extrasct/IC50 (%)	Fibrosarcoma cells (HT1080) Lung carcinoma cells (A549) Osteosarcoma cells (H2OS)	0.2–0.5	[108]
Red propolis	3-(4,5-dimethyl-2-thiazole)-2,5-diphenyl-2-H-tetrazolium bromide colorimetric assay MTT/ethanolic extracts/% ICG	Colon cancer cell lines (HCT116) Prostate cancer cell lines (PC3)	18.34–64.63	[57]
	Spectrophotometric plate reader method/ethanolic extracts/IC50 values/μg/mL	Ovarian cancer cells (OVCAR-8) Colon cancer cells (HCT-116) Leukemia cells (HL-60) Glioblastoma cells (SF-295)	23.63–27.08 19.92–30.19 4.80–8.75 13.67–18.47	[10]
	Spectrophotometric ELISA colorimetric assay/Ethanolic extracts /Absorbance units	Murine melanoma cellular strain (B16F10)	0.018–0.006	[9]
	Spectrophotometric microtitre plate reader Bio Assay/Ethanolic extracts and active fraction containing xanthochymol and formononetin/µg/mL	Melanoma tumour xenografts cell lines (HL-60, K562, RPMI8226, B16F10)	9.7–42.1	[109]

%ICG—Inhibition of cellular growth.
